# Constrast-enhanced computed tomography radiomics predicts CD27 expression and clinical prognosis in head and neck squamous cell carcinoma

**DOI:** 10.3389/fimmu.2022.1015436

**Published:** 2022-11-15

**Authors:** Fang Wang, Wenhao Zhang, Ying Chai, Hanshao Wang, Zhonglong Liu, Yue He

**Affiliations:** ^1^ Department of Oral Surgery, Shanghai Ninth People’s Hospital, Shanghai Jiao Tong University School of Medicine, College of Stomatology, Shanghai Jiao Tong University, National Center for Stomatology, National Clinical Research Center for Oral Diseases, Shanghai Key Laboratory of Stomatology, Shanghai Research Institute of Stomatology, Shanghai, China; ^2^ Department of Oromaxillofacial Head and Neck Oncology, Shanghai Ninth People’s Hospital, Shanghai Jiao Tong University School of Medicine, College of Stomatology, Shanghai Jiao Tong University, National Center for Stomatology, National Clinical Research Center for Oral Diseases, Shanghai Key Laboratory of Stomatology, Shanghai Research Institute of Stomatology, Shanghai, China

**Keywords:** enhanced-CT, radiomics, CD27, head and neck squamous cell carcinoma, clinical prognosis

## Abstract

**Objective:**

This study aimed to construct a radiomics model that predicts the expression level of *CD27* in patients with head and neck squamous cell carcinoma (HNSCC).

**Materials and methods:**

Genomic data and contrast-enhanced computed tomography (CT) images of patients with HNSCC were downloaded from the Cancer Genome Atlas and Cancer Imaging Archive for prognosis analysis, image feature extraction, and model construction. We explored the potential molecular mechanisms underlying *CD27* expression and its relationship with the immune microenvironment and predicted *CD27* mRNA expression in HNSCC tissues. Using non-invasive, CT-based radiomics technology, we generated a radiomics model and evaluated its correlation with the related genes and HNSCC prognosis.

**Results and conclusion:**

The expression level of *CD27* in HNSCC may significantly influence the prognosis of patients with HNSCC. Radiomics based on contrast-enhanced CT is potentially effective in predicting the expression level of *CD27*.

## Introduction

Head and neck squamous cell carcinoma (HNSCC) accounts for approximately 90% of all head and neck tumors (1). At present, the treatment of patients with HNSCC predominantly consists in surgery combined with radiotherapy and chemotherapy; however, the 5-year survival rate remains considerably low, and approximately half of the patients die within 5 years (2). The classical prognostic indicators of HNSCC, including clinical pathological features, presence of the human papillomavirus (HPV), and computed tomography (CT) and magnetic resonance imaging (MRI) findings, no longer satisfy the clinical requirements for precision medicine (3). Therefore, further investigation of novel prognostic markers and prognostic stratification of patients are necessary to provide new indicators for individualized precision treatment.

The T-cell activation antigen encoded by the *CD27* gene is a member of the tumor necrosis factor (TNF) receptor superfamily (4). The CD27 receptor is necessary for the production and long-term maintenance of T-cell immunity. It binds to the ligand CD70 and plays a key role in regulating B-cell activation and immunoglobulin synthesis (5). Furthermore, the CD27 protein transduces signals that lead to NF KappaB and MAPK8/JNK activation (6). The adaptor proteins TNF receptor-related factor (TRAF2) and TRAF5 have been shown to mediate the CD27 signaling process (7). The CD27 binding protein Siva is a pro-apoptotic protein that is considered to play an important role in CD27-induced cell apoptosis (8). The NCT01460134 phase I study evaluated the safety and dose in 56 patients with blood tumors and advanced solid tumors (including metastatic melanoma, renal cell carcinoma, prostate cancer, ovarian cancer, colorectal cancer, and non-small cell lung cancer). One of the 15 patients with metastatic renal cell carcinoma achieved partial remission, with a tumor reduction rate of 78% and progression-free survival of 2.3 years. Moreover, eight (14.29%) patients were stable for 3.8–47.3 months. Among 19 patients with advanced B-cell lymphoma who underwent intensive pretreatment, one achieved complete remission (9). To date, the ongoing phase I/II dose escalation and cohort expansion study (NCT02335918) that investigates combination therapy with nivolumab for different solid malignancies (including HNSCC) has only obtained results for colorectal and ovarian cancers. These results indicated that 5 of 49 patients (10%) with ovarian cancer achieved partial remission, while 19/49 (39%) achieved stability (10).

Radiomics is a high-throughput image sequencing technique that can yield several image feature parameters (11). It is a non-invasive, dynamic detection and quantitative-reaction technological tool for evaluating tumor characteristics. Radiomics technology has been used widely in clinical settings. Previous studies have demonstrated that radiomics can be used for the early diagnosis of HNSCC and classification into types as well as the evaluation of the tumor’s heterogeneity and microenvironment (12).

Therefore, this study aimed to (i) predict the mRNA expression of *CD27* in HNSCC tissues using non-invasive, CT-based radiomics technology; (ii) evaluate the correlation between the constructed radiomics model and the related genes and prognosis; and (iii) explore the potential molecular mechanism underlying the expression of *CD27* and its relationship with the immune microenvironment.

## Materials and Methods

### Sources of data and images

The medical imaging data (including the clinical and follow-up data) and transcriptome sequencing data (including the clinical and follow-up data) were obtained from The Cancer Imaging Archive (TCIA, https://www.cancerimagingarchive.net/) and the Cancer Genome Atlas (TCGA, https://portal.gdc.cancer.Gov/) databases, respectively, to determine the prognostic value of *CD27* expression as well as to construct a radiomics prediction model and determine its prognostic value. The R package “surfminer” was used to calculate the cutoff values.

### Analysis of differences between groups

UCSC Xena (https://xenabrowser.net/datapages/) RNA-Seq data in the “fragments per kilo base of transcript per million mapped fragments” (FPKM) format were processed uniformly *via* the coil process. The processing of the RNA-seq data in the FPKM format and a log_2_ transformation were performed to compare *CD27* expression between samples. The R package “ggplot2” was used for visualization.

### Survival analyses

Kaplan–Meier survival curve analysis was used to reveal changes in the survival rates in different groups, wherein the median survival time represented the survival time that corresponded to a survival rate of 50%. A log-rank test was used to test the significance of the survival rate in the groups.

### Univariate and multivariate Cox regression analysis

Cox proportional hazard models are potentially useful in examining the relationships between one or more research factors and survival outcomes. Therefore, a single-factor Cox regression was used in a comparison analysis to explore the influencing factors of overall survival (OS). Using a multifactor Cox regression, the eligibility of a factor as an independent influencing factor of OS was determined, and the role of multiple influencing factors was also explored. When the hazard ratio (HR) > 1, the independent variable was considered to be a risk factor. When the HR was < 1, the independent variable was considered as a protective factor. The R packages “survival” and “forest plot” were used in these analyses.

### Subgroup analysis and interaction test

An exploratory subgroup analysis was conducted using a univariate Cox regression to analyze the impact of the main variable, *CD27* expression, (high-expression group vs. low-expression group) on the prognosis of patients in each covariate subgroup. A likelihood ratio test was used to analyze the interaction between *CD27* expression and other covariates.

### Subtype analysis

Using the mesenchymal, basal, classical, and atypical subtypes of HNSC as groups, the differences in *CD27* expression values between the four groups were calculated. The subtype data was downloaded from PMID: 25631445, and the sample size was 272. The Kruskal–Wallis test was used to analyze the differences in *CD27* expression values between groups of different subtypes, and the Bonferroni correction was used to correct the differences between two groups.

### Single-cell sequencing analysis

The expression of *CD27* in immune cells was analyzed using the GSE103322 data set provided by the scTIME network (http://sctime.sklehabc.com/unicellular/home).

### Correlation analysis

Spearman’s rank correlation coefficient was used to analyze the correlation, and the results were displayed using a correlation heat map.

### Analysis of correlation between *CD27* expression and immune-cell infiltration

We uploaded the gene expression matrix of the HNSCC samples to the ImmuCellAI (http://bioinfo.life.hust.edu.cn/ImmuCellAI#!/) and CIBERSORTx (https://cibersortx.stanford.edu/) databases and measured immune-cell infiltration in each sample (13). Spearman’s rank correlation coefficient was used to analyze the correlation between the main variable *CD27* expression and immune cell infiltration. The R-package “limma” was used to analyze the differences in immune-cell infiltration between the *CD27* high- and low-expression groups, and the Wilcoxon test was used to draw the box diagram.

### Enrichment analysis of differentially-expressed genes

To further confirm the functions of potential targets, the data were analyzed using a function enrichment analysis. Gene Ontology (GO) is a widely-used tool for annotating functional genes, especially regarding their related molecular functions (MF), biological processes (BP), and cellular components (CC). The Kyoto Encyclopedia of Genes and Genomes (KEGG) is a widely-used database for storing information regarding genomes, biological pathways, diseases, and drugs. We visualized the top 10 significantly-enriched pathways obtained from the BP, CC, and MF enrichment analyses and the top 30 significantly-enriched pathways from the KEGG enrichment analysis. We used the R package “clusterprofiler” to conduct the GO (BP/CC/MF) and KEGG enrichment analyses, with a q-value filter < 0.05 as the filter condition.

### Screening of radiomics features

The parameters of 107 radiomics feature extracted using pyradiomics from 139 TCIA–TCGA intersection samples were standardized. The data were divided randomly into a training set and a validation set according to a 6:4 ratio using the R package “caret, cbcgrps.” The differences between the training and validation sets were analyzed.

Before modeling, features were screened through recursive feature elimination (RFE), whereby the predictive factors were sorted, and the less-important factors were eliminated in turn. The goal was to identify a subset of predictors that could be used to generate accurate models. We maintained model training, deleted *n* features of low importance after each training, subsequently retrained the new features to reacquire feature importance, and deleted *n* features of low importance again until the optimal feature subset was obtained.

### Construction of radiomics model

The support vector machine (SVM) algorithm uses support vectors to identify high-latitude hyperplanes as decision boundaries. Using the R package “caret,” the selected radiomics features were modeled using the SVM algorithm to predict gene expression.

### Evaluation of radiomics model and consistency evaluation

The radiomics model was used to evaluate the effectiveness of the models in the training and validation groups. The evaluation indexes included accuracy, specificity, sensitivity, positive predictive value, and negative predictive value. The x-axis of the receiver operating characteristic (ROC) curve represented the false-positive rate, while the y-axis represented the true-positive rate. The x-axis of the recall curve (PR) represented coverage (recall), that is, the true-positive rate, whereas the y-axis represented accuracy (precision). The calibration degree of the radiomics prediction model was evaluated by constructing the calibration-curve and using the Hosmer–Lemeshow goodness-of-fit testing method. The comprehensive performance of the image ensemble prediction model was quantified using the Brier Score. The clinical benefit of the radiomics prediction model was displayed *via* a decision curve analysis (DCA). The area under the curve (AUC) values of the training and validation sets were compared using the DeLong test to verify fitting.

The intraclass correlation coefficient (ICC) was used to evaluate the consistency of the radiomics features that were extracted based on the volume of interest (VOI) outlines obtained from two doctors. After one doctor sketched all the cases, 20 samples were selected randomly using the random number table method and sketched by another doctor, and the radiomics characteristics were then extracted.

### Time-dependent ROC curve

The probability value (radiomics score) of the prediction of the radiomics model, with a cutoff value of 0.565, was used to obtain the radiomics signature (RS) and combined with the clinical data. With respect to the survival analysis data (survival time and survival state), the disease status and factors were expected to change with time. We constructed different ROC curves according to varying time nodes, that is, time-dependent ROC curves, to illustrate the predictive ability of various factors at different time points. We also constructed the corresponding time-dependent ROC curves at different time nodes (12, 36, and 60 months) after HNSCC diagnosis and drew a time-based AUC curve to evaluate RS-expression differences in order to predict the survival of patients at different time points.

## Results

### Patient characteristics

A total of 483 patients with HNSCC from the TCGA database were included in the survival analysis and categorized into *CD27* high-expression (n = 242) and low-expression (n = 241) groups, with 1.2205156 as the cutoff value. The clinical information of the patients is shown in **Table 1**. Significant differences in the distribution of perineural invasion, radiotherapy, primary tumor site, and HPV status were noted between the *CD27* high- and low-expression groups.

**Table 1:** Patient characteristics.

Variables	Total (n = 483)	Low (n = 241)	High (n = 242)	*P*
Age (years)	n (%)			0.254
~59	211 (44)	112 (46)	99 (41)	
60~	272 (56)	129 (54)	143 (59)	
Gender	n (%)			0.129
Female	128 (27)	56 (23)	72 (30)	
Male	355 (73)	185 (77)	170 (70)	
Grade	n (%)			0.164
G1/G2	348 (72)	181 (75)	167 (69)	
G3/G4/GX	135 (28)	60 (25)	75 (31)	
Perineural invasion	n (%)			0.003
No	181 (37)	79 (33)	102 (42)	
Unknown	141 (29)	64 (27)	77 (32)	
Yes	161 (33)	98 (41)	63 (26)	
T stage	n (%)			0.269
T1/T2	173 (36)	80 (33)	93 (38)	
T3/T4/TX/Unknown	310 (64)	161 (67)	149 (62)	
N stage	n (%)			0.2
N0	164 (34)	89 (37)	75 (31)	
N1/N2/N3/NX/Unknown	319 (66)	152 (63)	167 (69)	
M stage	n (%)			0.413
M0	174 (36)	82 (34)	92 (38)	
M1/MX/Unknown	309 (64)	159 (66)	150 (62)	
Radiotherapy	n (%)			0.033
No	234 (48)	129 (54)	105 (43)	
Yes	249 (52)	112 (46)	137 (57)	
Chemotherapy	n (%)			0.13
No	322 (67)	169 (70)	153 (63)	
Yes	161 (33)	72 (30)	89 (37)	
Primary tumor site	n (%)			< 0.001
Larynx	109 (23)	42 (17)	67 (28)	
Oral cavity	297 (61)	174 (72)	123 (51)	
Oropharynx/Hypopharynx	77 (16)	25 (10)	52 (21)	
HPV status	n (%)			0.003
Negative	68 (14)	34 (14)	34 (14)	
Positive	30 (6)	6 (2)	24 (10)	
Unknown	385 (80)	201 (83)	184 (76)	

HPV, human papillomavirus.

### Association between gene expression level and clinical characteristics

**Figure 1:**
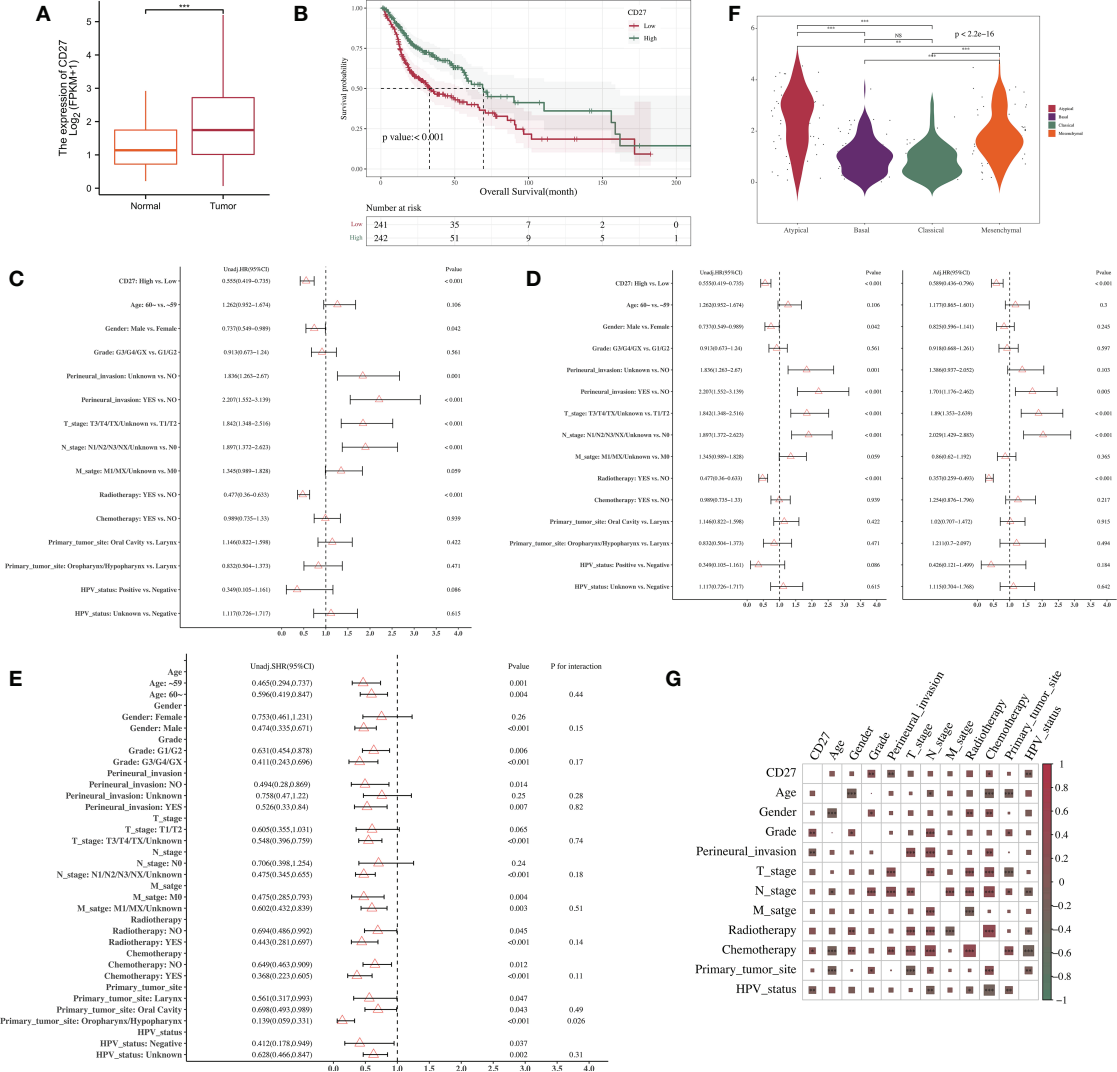
Association between gene expression level and clinical characteristics. **(A)** *CD27* expression in normal and tumor tissues. **(B)** Correlations between *CD27* expression and OS in HNSCC. **(C)** Univariate Cox regression analyses. **(D)** Multivariate Cox regression analyses. **(E)** Subgroup analysis and interaction test. **(F)** Subtype analysis. **(G)** Correlation analysis between *CD27* expression and clinical parameters of HNSCC. **p < 0.01, ***p < 0.001, NS, no significance.

We found the *CD27* expression in tumors to be higher than that in normal tissue, and the difference was statistically significant (**Figure 1A**). The median survival times in the *CD27* low- and high-expression groups were 32.93 (95% confidence interval [CI], 25.43–58.26) and 69.43 (95% CI, 57.73–158.66) months, respectively. The Kaplan–Meier curve analysis demonstrated that higher *CD27* expression levels were associated with OS improvement (**Figure 1B**). In addition, the T and N cancer stages, perineural invasion, and radiotherapy were also associated significantly with OS (**Figures S1A–D**). Furthermore, the univariate analysis showed that high *CD27* expression (HR = 0.555; 95% CI, 0.419–0.735, *P* < 0.001), male, and radiotherapy were protective factors for OS (**Figure 1C**). After multivariate adjustment, the multivariate analysis showed that high *CD27* expression (HR = 0.589; 95% CI, 0.436–0.796, *P* < 0.001) and radiotherapy were protective factors for OS (**Figure 1D**). Within the primary-tumor-site subgroup, the subgroup analysis revealed that a *CD27* increase was a protective factor for OS. The *P* value for the interaction test was 0.026, suggesting a significant interaction between *CD27* and the different primary tumor sites (**Figure 1E**). Besides, the difference in the expression values of *CD27* between different subtypes was statistically significant (*P* < 0.001). After pairwise comparison, there was no statistical difference in *CD27* expression between the basal and classical groups, but there were significant differences in *CD27* expression between the atypical and basal groups and the basal and mesenchymal groups (*P* < 0.001) (**Figure 1F**). Moreover, the *CD27* expression value in the atypical group was higher than that in the basal, classical, and mesenchymal groups. Finally, the main variable, *CD27* expression, showed a significant correlation with the tumor grade, perineural invasion, chemotherapy, and the HPV status (**Figure 1G**).

### *CD27* expression correlates with HNSCC immune-cell infiltration

**Figure 2:**
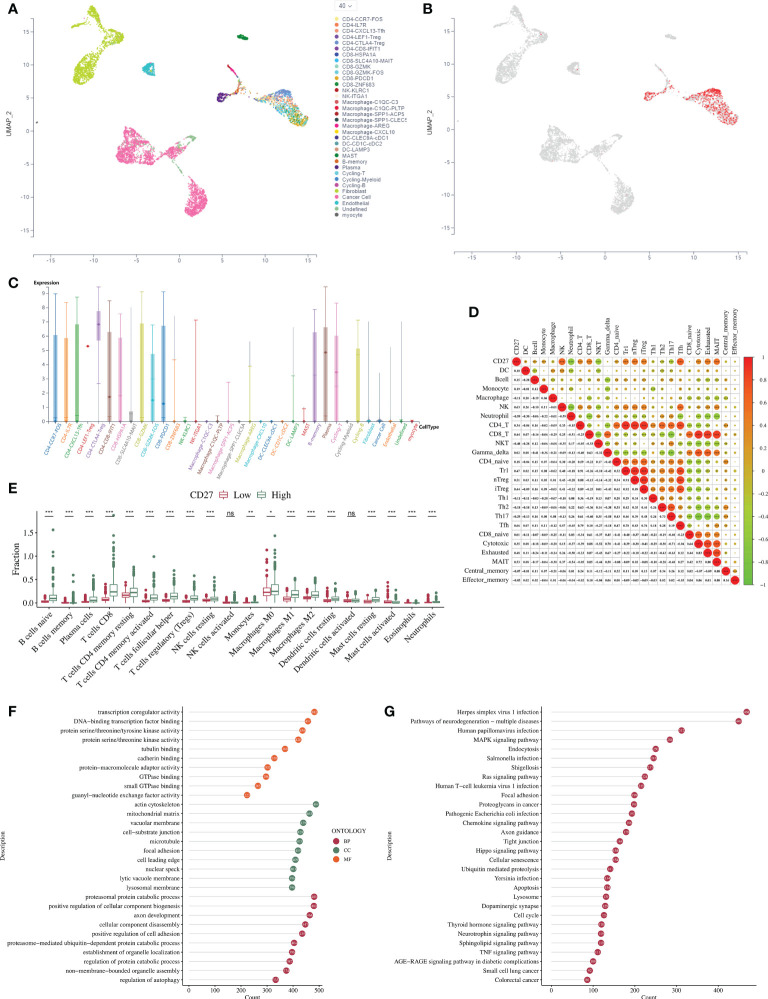
Analysis of the immune-cell infiltration and the DEGs associated with *CD27* expression in HNSCC. **(A)** Clustering of 40 types of immune cells. **(B)** Immune cells exhibiting *CD27* expression. **(C)** Expression and distribution of *CD27* in 33 types of immune cells. **(D)** Correlations between *CD27* expression and immune-cell infiltration in HNSCC in the ImmuCellAI database. **(E)** Correlations between *CD27* expression and immune-cell infiltration in HNSCC in the CIBERSORTx database. **(F)** GO enrichment analysis. **(G)** KEGG enrichment analysis. *p < 0.05, **p < 0.01, ***p < 0.001, NS, no significance.

We analyzed single-cell sequencing data on HNSCC provided by the scTIME network. We visualized the clustering of 40 immune cells (**Figure 2A**) and the distribution of *CD27* expression in 33 immune cells (**Figures 2B, C**). Single-cell RNA sequencing analysis showed that *CD27* was expressed in CD4-CCR7-FOS, CD4-LEF1 Treg, cycling T cells, and other immune cells. Besides, the correlation analysis showed that *CD27* expression had a significant correlation with cytotoxic T cells, depleted T cells, and other activated T cells (*P* < 0.001) (**Figure 2D**). Moreover, the degree of macrophage (M0 cell) infiltration was significantly elevated in the *CD27* high-expression group (*P* < 0.05), while the degree of infiltration of activated natural killer (NK) cells was not significantly different between the two groups (*P* > 0.05) (**Figure 2E**).

### Analysis of differentially-expressed genes (DEGs) in HNSCC associated with high and low *CD27* expression

The GO enrichment analysis revealed that DEGs related to DNA-binding, transcription-factor binding, and transcriptional auxiliary regulatory activity were enriched in the *CD27* high-expression group compared to the low-expression group (**Figure 2F**). The KEGG enrichment analysis revealed that DEGs were significantly enriched in the MAPK signaling pathway, cell cycle, and RAS signaling pathway in the *CD27* high-expression group compared to the low-expression group (**Figure 2G**).

### Clinicopathological characteristics of radiomics participants

**Figure 3:**
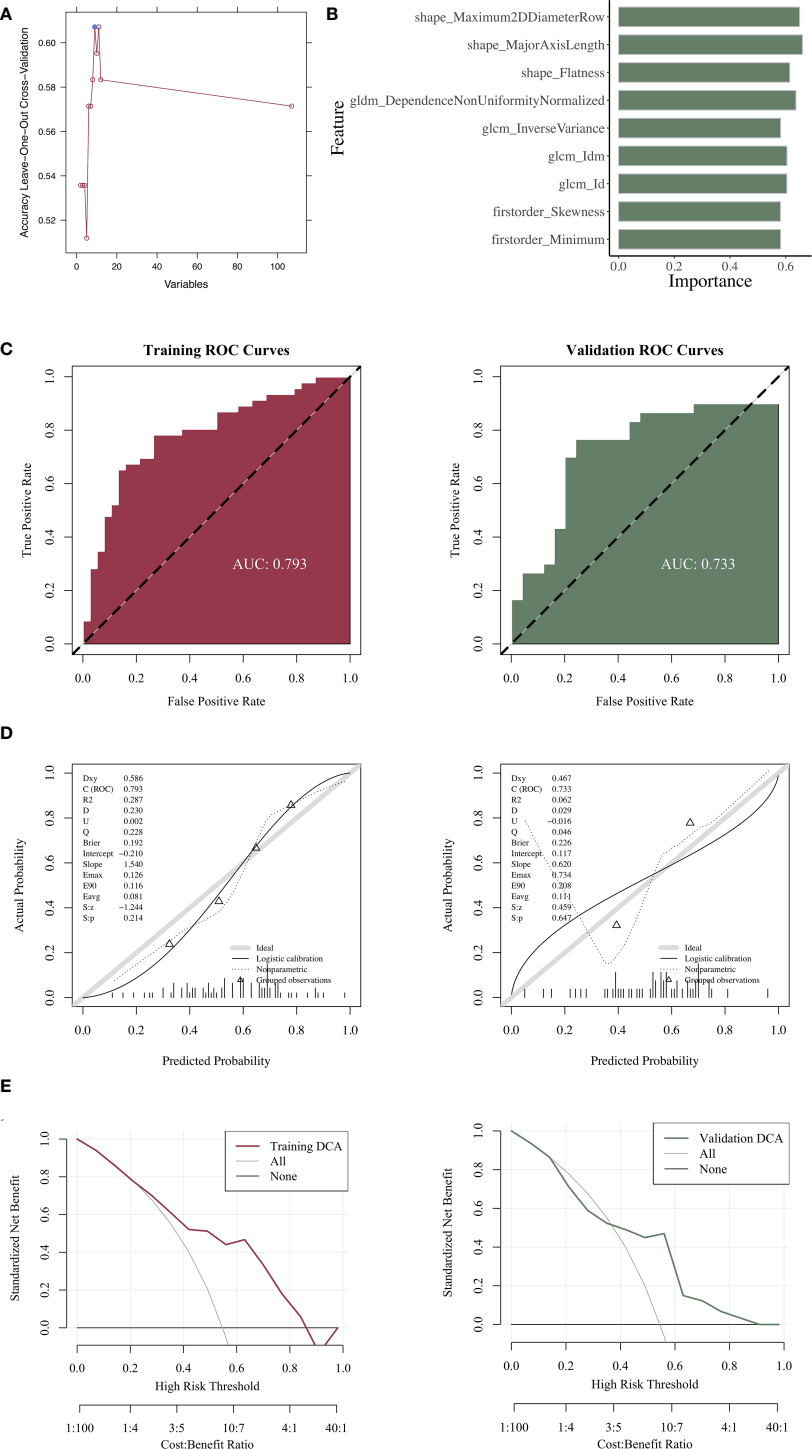
Construction and evaluation of the radiomics models. **(A)** Radiomics features with statistical differences. **(B)** Importance of the selected features in the model. **(C)** ROC curve analysis of the radiomics model. **(D)** Calibration-curve analysis of the radiomics model. **(E)** Hosmer–Lemeshow goodness-of-fit testing of the radiomics model.

We divided the overall data set and obtained 84 training sets and 55 verification sets (**Table 2**). The *P* value of each variable in the analysis of differences between groups was > 0.05, indicating that the baseline status of the patients in the training and validation sets was consistent and comparable between groups. Nine radiomics features were screened out (**Figure 3A**).

**Table 2:** Data set division.

Variables	Total (n = 139)	Train (n = 84)	Validation (n = 55)	*P*
CD27	n (%)			1
Low	63 (45)	38 (45)	25 (45)	
High	76 (55)	46 (55)	30 (55)	
Gender	n (%)			0.219
Female	34 (24)	17 (20)	17 (31)	
Male	105 (76)	67 (80)	38 (69)	
Age	n (%)			0.526
~59	64 (46)	41 (49)	23 (42)	
60~	75 (54)	43 (51)	32 (58)	
HPV_status	n (%)			0.422
Negative	15 (11)	11 (13)	4 (7)	
Positive/Unknown	124 (89)	73 (87)	51 (93)	
Perineural_invasion	n (%)			0.373
NO	48 (35)	28 (33)	20 (36)	
Unknown	49 (35)	27 (32)	22 (40)	
YES	42 (30)	29 (35)	13 (24)	
Primary_tumor_site	n (%)			0.5
Larynx	34 (24)	19 (23)	15 (27)	
Oral Cavity	84 (60)	50 (60)	34 (62)	
Oropharynx/Hypopharynx	21 (15)	15 (18)	6 (11)	
Grade	n (%)			0.424
G1/G2	97 (70)	56 (67)	41 (75)	
G3/G4/GX	42 (30)	28 (33)	14 (25)	
T_stage	n (%)			0.739
T1/T2	42 (30)	24 (29)	18 (33)	
T3/T4/TX/Unknown	97 (70)	60 (71)	37 (67)	
N_stage	n (%)			0.506
N0	54 (39)	35 (42)	19 (35)	
N1/N2/N3/NX/Unknown	85 (61)	49 (58)	36 (65)	
M_stage	n (%)			0.027
M0	66 (47)	33 (39)	33 (60)	
M1/MX/Unknown	73 (53)	51 (61)	22 (40)	
Radiotherapy	n (%)			0.404
NO	68 (49)	44 (52)	24 (44)	
YES	71 (51)	40 (48)	31 (56)	
Chemotherapy	n (%)			0.855
NO	96 (69)	59 (70)	37 (67)	
YES	43 (31)	25 (30)	18 (33)	
OS	n (%)			0.335
0	88 (63)	50 (60)	38 (69)	
1	51 (37)	34 (40)	17 (31)	1

HPV, human papillomavirus; OS, overall survival.

### Construction and evaluation of radiomics models

We constructed a radiomics model and analyzed the importance of the selected features (**Figure 3B**). After the evaluation, the radiomics model exhibited a favorable predictive ability, as demonstrated by the ROC curve. The model’s AUC values in the training and validation sets were 0.793 and 0.733, respectively (**Figure 3C**). The calibration-curve analysis (**Figure 3D**) and Hosmer–Lemeshow goodness-of-fit testing (**Figure 3E**) revealed that the prediction probability of the radiomics prediction model for determining the level of genetic expression was consistent with the true value (*P* > 0.05). The DCA model displayed clinical practicability. When comparing the AUC values, no statistical difference was noted between the validation and training sets (*P* = 0.496), indicating that the model had a favorable fit. In addition, the ICC values of the radiomics features included in the model were all above 0.85, indicating that these radiomics features had a favorable consistency (**Table 3**).

**Table 3:** Radiomics features.

Item	Impotence
original_shape_Maximum2DDiameterRow	0.988477126
original_firstorder_Minimum	0.923561705
original_gldm_DependenceNonUniformityNormalized	0.996929778
original_shape_Flatness	0.990406861
original_glcm_InverseVariance	0.992422513
original_glcm_Idm	0.989387306
original_shape_MajorAxisLength	0.995909019
original_firstorder_Skewness	0.881504566
original_glcm_Id	0.989876579

### Association between RS and clinical characteristics

**Figure 4:**
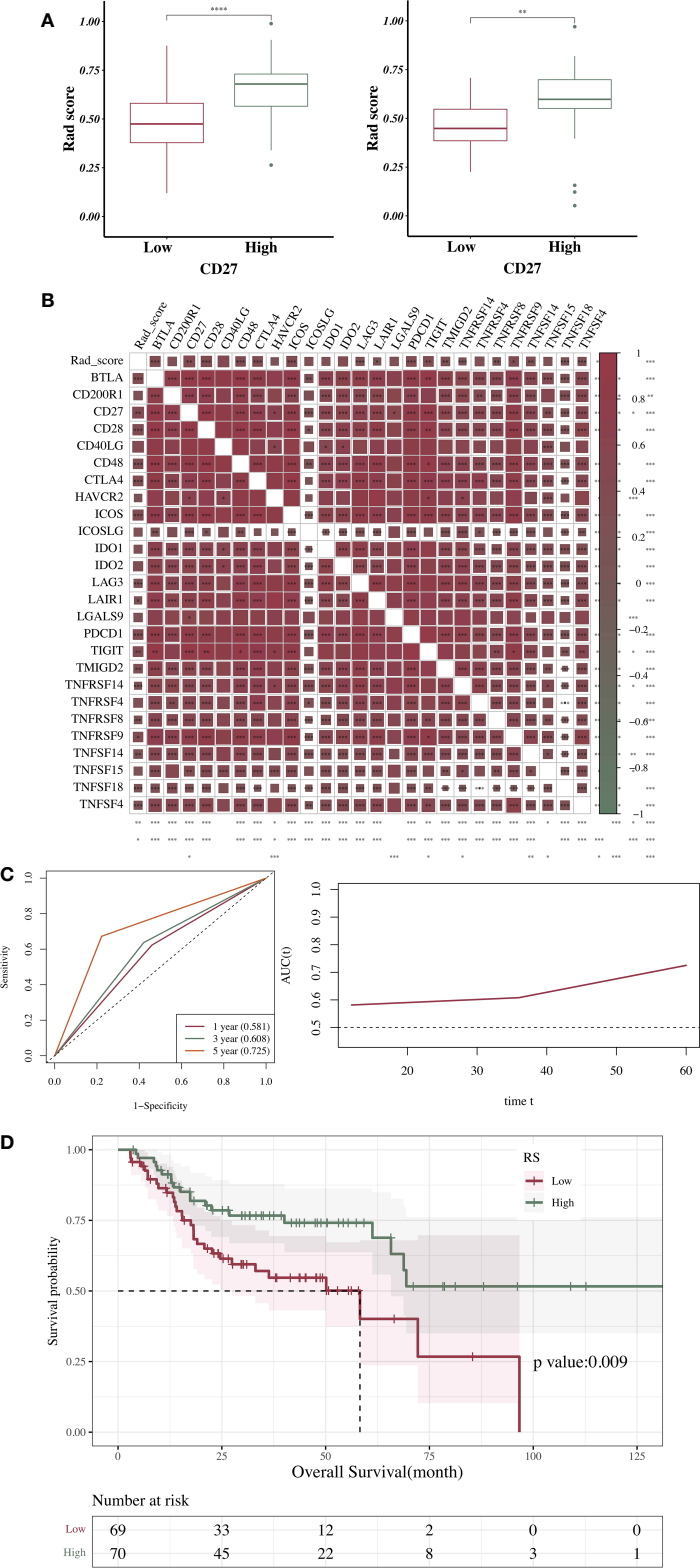
Association between RS and clinical characteristics. **(A)** Association between RS and *CD27* expression. **(B)** Correlation analysis of RS, *CD27* expression, and expression of immune genes expression. **(C)** Time-dependent ROC curve obtained according to the AUC value of the main variable at each time point (12, 36, and 60 months). **(D)** Correlations between RS and OS in HNSCC. *p < 0.05, **p < 0.01, ***p < 0.001, ****p < 0.0001.

The RS distribution in the training and validation sets was significantly different between the *CD27* high- and low-expression groups (p < 0.05). In the *CD27* high-expression group, the RS value was high (**Figure 4A**). The RS exhibited a significant positive correlation with the model’s prediction of *CD27* expression, with a correlation coefficient of 0.420 (*P* < 0.001). The correlation between the RS value and the expression of immune-related genes was similar to that between *CD27* expression and the expression of immune-related genes, with both correlations being positive and statistically significant (**Figure 4B**). A total of 139 patients with HNSCC from the TCGA database were included in the survival analysis and were divided into the RS for high *CD27* expression (n = 70) and low *CD27* expression (n = 69) groups. The clinical information of the patients is shown in **Table S1**. As shown by the ROC curve, the AUC value for the ability of *CD27* expression, as determined by the RS, to predict OS prognosis (60 months) was 0.725. The AUC value increased with time (**Figure 4C**). Finally, the median survival time in the *CD27* low-expression group, as determined by the RS, was 58.27 months. In contrast, the median survival time in the *CD27* high-expression group, as determined by the RS, was longer than the follow-up period, demonstrating that a higher RS value was related to OS improvement (**Figure 4D**).

## Discussion

Since the classical approaches for identifying prognostic indicators in HNSCC, such as CT and MRI, no longer satisfy the clinical requirements for precision medicine (14), identification of novel prognostic markers and stratification of patients based on prognosis are warranted to provide new indicators for individualized precision treatment (15). On this premise, this study established *CD27* expression as a potentially significant prognostic indicator in patients with HNSCC.

*CD27*, a member of the TNF receptor superfamily, is structurally expressed on immature T cells, memory B cells, NK cells, hematopoietic stem cells, and progenitor cells. CD27 is a transmembrane phosphoglycoprotein expressed on CD4+ and CD8 + T cells (5). *CD27* expression increases when T cells are activated, as well as during apoptosis. After activation, CD27 takes a soluble form (sCD27). CD70 (also known as CD27L) is the sole ligand of CD27. After binding with CD70, CD27 can bind to TRAF to produce intracellular signals that may potentially enhance the survival and activation of T cells, B cells, and NK cells through the activation of TRAF2 and TRAF5 signals and the NF-κB pathway (16). CD27–CD70 interaction is strictly regulated to prevent overexpression and subsequent lymphocyte overactivation (17). In this study, we found that *CD27* expression was significantly and positively correlated with T- and B-cell infiltration, thus further corroborating the above view. Besides, the correlation analysis showed that *CD27* expression had a weak correlation with naive CD8+ T cells, while it had a significant correlation with cytotoxic T cells, depleted T cells, and other activated T cells.

Recently, with the development of T-cell therapy with immune checkpoint inhibitors and other advances in treating tumors, the costimulatory mechanisms underlying T-cell activation and proliferation have drawn increasing attention (18, 19). Costimulatory activation of the CD27–CD70 axis has been has been harnessed as a treatment for cancers made of various tumor cell types (20, 21). Therefore, activation of the CD27–CD70 axis as a novel, future therapeutic target demonstrates great prospects.

Radiomics can be used to predict prognosis in several diseases (22). In some studies, the treatment response of patients with HNSCC undergoing radical radiotherapy was predicted based on quantitative ultrasound radiomics with an accuracy > 90% (23). The SVM model can accurately identify patients at high risk of disease recurrence, thus having a great impact on survival rate (24). Additional studies have also compared several models, including logistic regression, random forest, Naïve Bayes, SVM, AdaBoost, and neural network models, in predicting occult neck lymph node metastasis in early oral tongue squamous cell carcinoma based on preoperative MRI texture features (25–28). These studies have demonstrated that Naïve Bayes offers the best performance, with the node status correctly identified in 74.1% of patients. All the above studies underscore the potential of radiomics in clinical treatment.

Radiomics has shown great potential in predicting tumor prognosis and evaluating treatment effect, but there are still some limitations. For example, there is a lack of multicenter prospective radiomics research to guide clinical practice. The reason is that there is no gold standard yet for radiomics research methods and the repeatability of radiomics features needs to be further improved. In addition, image reconstruction algorithms, preprocessing methods, individual differences, and feature extraction algorithms can affect the stability and repeatability of the radiomics features. At present, radiomics is still in its infancy. Solving the repeatability issue is the key for future research, and will further promote individualized, precise treatment in cancer.

This study aimed to predict *CD27* expression in HNSCC and determine its clinical prognostic value based on an enhanced-contrast CT radiomics model. For example, CD27 in TCGA-BA-5152 was measured to be highly expressed. As determined by the radiomics model, the radiomics score was 0.632 (the threshold determined by the Joden index of the ROC curve was 0.62), predicting that CD27 was highly expressed. In addition, as a costimulatory T-cell receptor, *CD27* is closely related to the prognosis of patients with HNSCC (29). In this study, we found *CD27* to be correlated with tumor grade. The Kaplan–Meier curve revealed that a low molecular expression was associated with the deterioration of survival. A multivariate analysis revealed that a high *CD27* expression was a protective factor for OS. Most patients with HNSCC who reached the advanced stage at diagnosis exhibited no obvious precancerous lesions clinically. HNSCC possesses significant heterogeneity, and increasing prognostic information may be helpful in clinical decision-making. In this study, we constructed a model for selected imaging characteristics in order to predict the expression level of *CD27* non-invasively. This may provide a novel diagnostic tool to faciliate the clinical application of immunotherapy.

Contrast-enhanced CT may increase the difference in density between lesions and adjacent normal tissues through the injection of contrast agent, thereby improving the lesions’ display rate (30); nonetheless, contrast-enhanced CT lacks objectivity and is non-quantitative. On the other hand, radiomics can mine high-throughput features from the images, recognize deep information that cannot be obtained directly with the naked eye, and conduct quantitative analysis. In previous studies, it was possible to distinguish the phenotypes of metastatic colorectal cancer by evaluating their imaging characteristics (31). One study that used positron emission tomography imaging to predict disease-free survival and OS in patients with gastric cancer found that it exhibited a predictive function superior to that of the tumor–node–metastasis staging system or tumor markers (32).

Machine learning is a specific field of artificial intelligence, which can be broadly defined as a computing algorithm/method that uses data to improve performance or make accurate predictions (33). The six most commonly-used machine learning classifiers are Logical Regression, Naive Bayes, SVM, Decision Tree, Neural Networks, and Deep learning (33). In this study, an RFE algorithm was used to identify the optimal feature set (34), and the SVM model was established to predict *CD27* expression. This prediction model exhibited a favorable consistency between its predictive probability and the true value of gene expression. DCA revealed that the model had high clinical practicability. No statistical differences in the AUCs of the training and validation sets were observed, and the model exhibited a favorable model fit. When applying the DeLong test to verify the fitting of the training and validation sets, the AUC value of the SVM model was found to exhibit no statistical differences between the training and validation sets, and the model exhibited a favorable prediction efficiency. Further analysis found the probability of predicting the gene-expression-level Rad score to be positively correlated with *CD27* expression. The Wilcoxon test revealed significant Rad-score differences between the *CD27* high- and low-expression groups, and both the training and validation sets. In addition, Kaplan–Meier curve analysis revealed an association between high Rad scores and improved OS, indicating that our radiomics-based prediction model had a favorable efficiency in predicting *CD27* gene expression and potentially offered substantial value in guiding clinical prognosis prediction.

## Conclusion

Our results showed that a significant correlation existed between *CD27* expression and the prognosis of HNSCC. Based on our contrast-enhanced CT radiomics model, the expression level of *CD27* can be predicted effectively. This prediction model, which was based on radiomics characteristics, exhibited favorable stability and diagnostic efficiency.

## Data availability statement

Publicly available datasets were analyzed in this study. This data can be found here: The medical imaging data (including the clinical and follow-up data) and transcriptome sequencing data (including the clinical and follow-up data) were obtained from The Cancer Imaging Archive (TCIA, https://www.cancerimagingarchive.net/) and the Cancer Genome Atlas (TCGA, https://portal.gdc.cancer.Gov/) databases.

## Author contributions

YH designed the experiments. FW, WZ, and YC collected the data. HW and ZL performed the calculations. FW and WZ wrote the main manuscript text, and all the authors approved the manuscript. All authors contributed to the article and approved the submitted version.

## Funding

This study was supported by the Fundamental Research Program of the Ninth People’s Hospital affiliated to the Shanghai Jiao Tong University School of Medicine (JYZZ158); The National Natural Science Foundation of China (82173451 and 81900969); Special Research on Medical Innovation of Shanghai Municipal Commission of Science and Technology (21Y11903500); Clinical Research Booster Program of Ninth People’s Hospital, Shanghai Jiao Tong University School of Medicine (JYLJ201903); Shanghai Jiaotong University Medical-Engineering Cross Research Fund (YG2022QN050), Science and Technology Commission of Shanghai Funding/Supporting (16411960900); Shanghai Municipal Science Research Project (SHDC12017101), Shanghai Summit & Plateau Disciplines and General project of Shanghai Health and Family Planning Commission (201740168).

## Conflict of interest

The authors declare that the research was conducted in the absence of any commercial or financial relationships that could be construed as a potential conflict of interest.

## Publisher’s note

All claims expressed in this article are solely those of the authors and do not necessarily represent those of their affiliated organizations, or those of the publisher, the editors and the reviewers. Any product that may be evaluated in this article, or claim that may be made by its manufacturer, is not guaranteed or endorsed by the publisher.

## Supplementary material

Supplementary Figure 1 Correlations between the T **(A)** and N **(B)** stages, perineural invasion **(C)**, radiotherapy **(D)**, and OS in HNSCC.
