# Supplementary figures and images for: Constrast-enhanced computed tomography radiomics predicts CD27 expression and clinical prognosis in head and neck squamous cell carcinoma
Source: Front Immunol. 2022 Nov 15;13:1015436. doi: 10.3389/fimmu.2022.1015436 (PMC9705340; doi:10.3389/fimmu.2022.1015436)

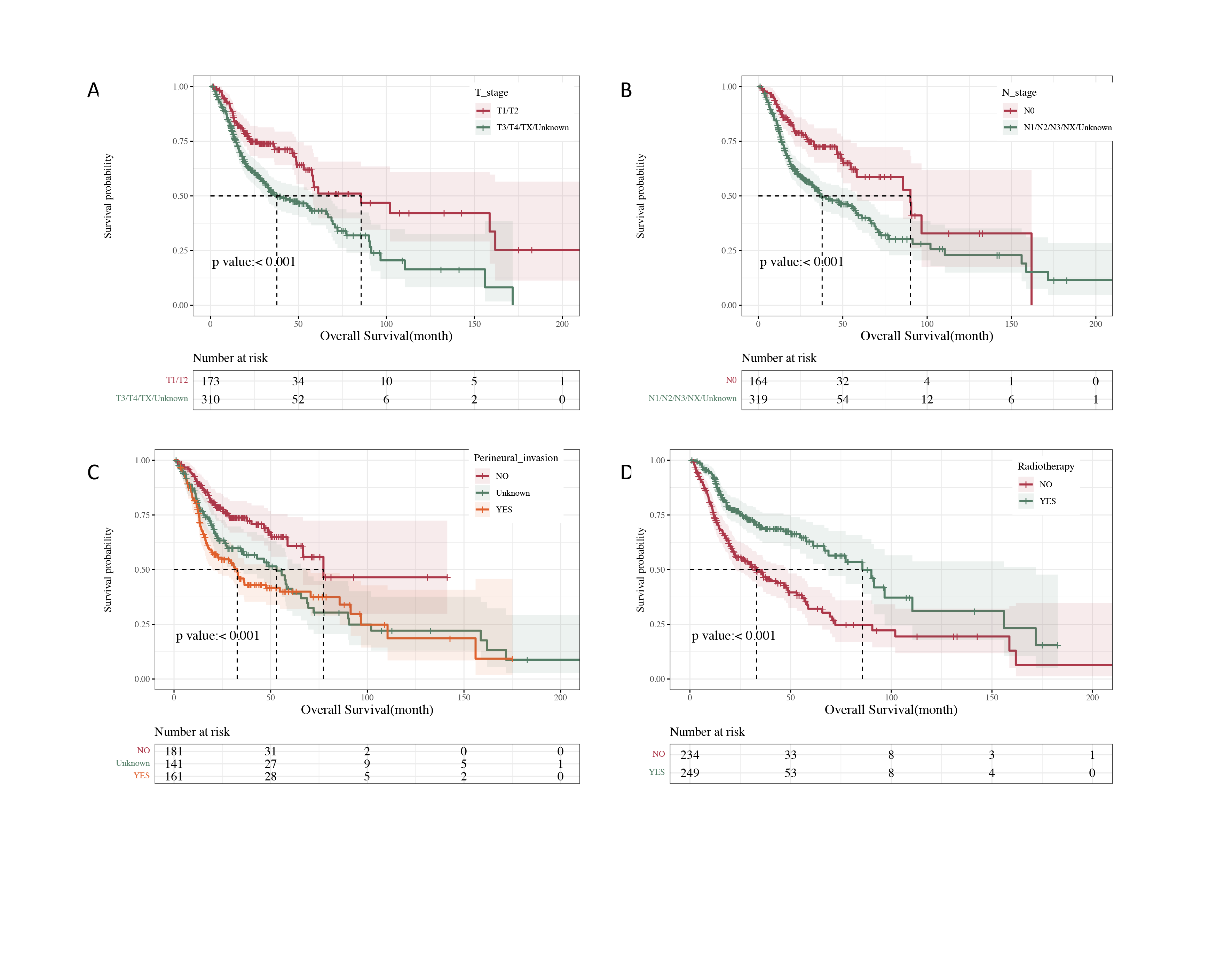

Supplement: Supplementary Figure 1 — Correlations between the T (A) and N (B) stages, perineural invasion (C), radiotherapy (D), and OS in HNSCC. [file Image_1.tif]
