# Supplementary material for: Constrast-enhanced computed tomography radiomics predicts CD27 expression and clinical prognosis in head and neck squamous cell carcinoma
Source: Front Immunol. 2022 Nov 15;13:1015436. doi: 10.3389/fimmu.2022.1015436 (PMC9705340; doi:10.3389/fimmu.2022.1015436)
Supplement: Supplementary file 2 [file Table_1.doc]

Table S1 Patient characteristics in the radiology scores high and low groups

| Variables | | | Total (n = 139) | | Low (n = 69) | High (n = 70) | | | *P* | |
| --- | --- | --- | --- | --- | --- | --- | --- | --- | --- | --- |
|  | Gender | n (%) | |  | | |  | 0.522 | |  |
|  | Female | 34 (24) | | 19 (28) | | | 15 (21) |  | |  |
|  | Male | 105 (76) | | 50 (72) | | | 55 (79) |  | |  |
|  | Age (years) | n (%) | |  | | |  | 0.44 | |  |
|  | ~59 | 64 (46) | | 29 (42) | | | 35 (50) |  | |  |
|  | 60~ | 75 (54) | | 40 (58) | | | 35 (50) |  | |  |
|  | HPV status | n (%) | |  | | |  | 0.261 | |  |
|  | Negative | 15 (11) | | 10 (14) | | | 5 (7) |  | |  |
|  | Positive/Unknown | 124 (89) | | 59 (86) | | | 65 (93) |  | |  |
|  | Perineural invasion | n (%) | |  | | |  | 0.909 | |  |
|  | No | 48 (35) | | 23 (33) | | | 25 (36) |  | |  |
|  | Unknown | 49 (35) | | 24 (35) | | | 25 (36) |  | |  |
|  | Yes | 42 (30) | | 22 (32) | | | 20 (29) |  | |  |
|  | Primary tumor site | n (%) | |  | | |  | 0.694 | |  |
|  | Larynx | 34 (24) | | 16 (23) | | | 18 (26) |  | |  |
|  | Oral cavity | 84 (60) | | 44 (64) | | | 40 (57) |  | |  |
|  | Oropharynx/Hypopharynx | 21 (15) | | 9 (13) | | | 12 (17) |  | |  |
|  | Grade | n (%) | |  | | |  | 1 | |  |
|  | G1/G2 | 97 (70) | | 48 (70) | | | 49 (70) |  | |  |
|  | G3/G4/GX | 42 (30) | | 21 (30) | | | 21 (30) |  | |  |
|  | T stage | n (%) | |  | | |  | 0.216 | |  |
|  | T1/T2 | 42 (30) | | 17 (25) | | | 25 (36) |  | |  |
|  | T3/T4/TX/Unknown | 97 (70) | | 52 (75) | | | 45 (64) |  | |  |
|  | N stage | n (%) | |  | | |  | 0.649 | |  |
|  | N0 | 54 (39) | | 25 (36) | | | 29 (41) |  | |  |
|  | N1/N2/N3/NX/Unknown | 85 (61) | | 44 (64) | | | 41 (59) |  | |  |
|  | M stage | n (%) | |  | | |  | 0.555 | |  |
|  | M0 | 66 (47) | | 35 (51) | | | 31 (44) |  | |  |
|  | M1/MX/Unknown | 73 (53) | | 34 (49) | | | 39 (56) |  | |  |
|  | Radiotherapy | n (%) | |  | | |  | 0.67 | |  |
|  | No | 68 (49) | | 32 (46) | | | 36 (51) |  | |  |
|  | Yes | 71 (51) | | 37 (54) | | | 34 (49) |  | |  |
|  | Chemotherapy | n (%) | |  | | |  | 1 | |  |
|  | No | 96 (69) | | 48 (70) | | | 48 (69) |  | |  |
|  | Yes | 43 (31) | | 21 (30) | | | 22 (31) |  | |  |

Abbreviation: HPV, human papillomavirus.
